# Nivolumab plus ipilimumab versus nivolumab in individuals with treatment-naive programmed death-ligand 1 positive metastatic soft tissue sarcomas: a multicentre retrospective study

**DOI:** 10.1186/s12885-021-07843-3

**Published:** 2021-02-02

**Authors:** Yaolin Chen, Xiangzhen Liu, Jijun Liu, Donghua Liang, Mingdong Zhao, Weiguang Yu, Pengfei Chen

**Affiliations:** 1 Department of Orthopaedics, Henan Provincial People’s Hospital, Department of Orthopaedics of Central China Fuwai Hospital,Central China Fuwai Hospital of Zhengzhou University, No. 1, Fuwai Avenue, Zhengdong New District, Zhengzhou, 450003 China; 2 Department of Oral and Maxillofacial Surgery, The First Affiliated Hospital, Sun Yat-sen University, No. 58, Zhongshan 2nd Road, Yuexiu District, Guangzhou, 510080 China; 3 Department of Orthopaedics, Jinshan Hospital, Fudan University, No. 1508, Longhang Road, Jinshan District, Shanghai, 201508 China; 4 Department of Orthopaedics, The First Affiliated Hospital, Sun Yat-sen University, No. 58, Zhongshan 2nd Road, Yuexiu District, Guangzhou, 510080 China

**Keywords:** Overall survival, Adverse events, Metastatic, Sarcoma, Treatment

## Abstract

**Background:**

Currently, the choice of treatment for individuals with metastatic soft tissue sarcomas (MSTS) presents a significant challenge to clinicians. The aim of this retrospective study was to assess the efficacy and safety of nivolumab plus ipilimumab (NPI) versus nivolumab alone (NIV) in individuals with treatment-naive programmed death-ligand 1 (PD-L1) positive MSTS.

**Methods:**

Prospectively maintained databases were reviewed from 2013 to 2018 to assess individuals with treatment-naive PD-L1 MSTS who received NPI (nivolumab 3 mg/kg and ipilimumab 1 mg/kg every 3 weeks for 4 doses followed by nivolumab 3 mg/kg every 2 weeks) or NIV (3 mg/kg every 2 weeks) until disease progression, withdrawal, unendurable [AEs], or death. The co-primary endpoints were overall survival (OS) and progression-free survival (PFS).

**Results:**

The median follow-up was 16.0 months (IQR 14.4–18.5) after targeted intervention. The median OS was 12.2 months (95% confidence interval [CI], 6.1–13.7) and 9.2 months (95% CI, 4.2–11.5) for the NPI and NIV groups, respectively (hazard ratio [HR] 0.49, 95% CI, 0.33–0.73; *p*=0.0002); the median PFS was 4.1 months (95% CI, 3.2–4.5) and 2.2 months (95% CI, 1.1–3.4) for the NPI and NIV groups, respectively (HR 0.51, 95% CI, 0.36–0.71; *p*< 0.0001). Key grade 3–5 AEs occurred more frequently in the NPI group than in the NIV group (94 [72.9%] for NPI vs. 35 [27.1%], *p*< 0.001).

**Conclusions:**

For treatment-naive PD-L1 positive MSTS, NPI seems to be less tolerated but has a greater survival advantage than NIV as the primary therapy.

## Background

Soft tissue sarcoma (STS) is a heterogeneous malignant tumour derived from mesenchymal cells that displays a heterogeneous mix of clinical and pathologic characteristics and is largely resistant to conventional therapies [1–3]. Evidence-based statistics [4, 5] have indicated that the majority of individuals with STS tend to develop recurrent or metastatic disease and are associated with poor outcomes. Few, if any, chemotherapy regimens, either alone or in combination, can reverse this situation [6]. For individuals with treatment-naive metastatic STS (MSTS), several approved chemotherapy regimens (i.e., doxorubicin, either alone or in combination) seem to have similar effects, with a low response rate, progression-free survival (PFS) of nearly 0.5 years and overall survival (OS) of 1–1.5 years [7, 8]. Except for first-line chemotherapeutics, all other chemotherapeutics that have been approved, to a certain extent, have improved OS in the absence of progression or metastasis of the STS [9, 10]. However, for MSTS, the median PFS tends to be approximately 4 months, and OS from a diagnosis of MSTS is less than 14 months [7, 11]. Management of such individuals is still a challenge, and a poor prognosis seems to be inevitable [5].

Nivolumab, a fully human immunoglobulin G4PD-1 immune checkpoint-blocking antibody, explicitly binds to programmed death 1(PD-1) and interrupts negative signalling to restore T-cell anti-tumour function, which leads to improved survival and a promising safety profile in individuals with specific progressed solid tumours involving STS [7, 11, 12]. Findings from a recent randomized clinical trial [7] demonstrated that nivolumab, alone or combined with ipilimumab (a cytotoxic T-lymphocyte antigen-4 checkpoint inhibitor), had promising efficacy for specified sarcoma subtypes, with a controllable safety profile consistent with current confirmed alternatives. However, there is a paucity of published information regarding the utilization of nivolumab and/or ipilimumab in treatment-naive programmed death-ligand 1 (PD-L1) positive MSTS individuals [11]. We report herein a retrospective study assessing the efficacy and safety of nivolumab alone or combined with ipilimumab in this setting.

## Methods

### Study design and patient eligibility

Clinical data of treatment-naive PD-L1 MSTS patients were identified retrospectively from a registry database involving three medical institutions from January 1, 2013 to December 31, 2018. The cohort consisted of 214 individuals with histologically confirmed, unresectable, treatment-naive MSTS who were treated with nivolumab plus ipilimumab (NPI: nivolumab 3 mg/kg and ipilimumab 1 mg/kg every 3 weeks for 4 doses followed by nivolumab 3 mg/kg every 2 weeks) or nivolumab alone (NIV: nivolumab 3 mg/kg every 2 weeks) until disease progression, withdrawal, unendurable AEs, or death [7]. The key inclusion criteria were as follows: age ≥ 16 years; a histologically definite diagnosis of STS with at least one measurable lesion per Response Evaluation Criteria in Solid Tumours (RECIST) v1.1 [8]; PD-L1 positive STS in the primary tissue; untreated MSTS; acceptable organ function (i.e., heart, liver, and kidney); and an Eastern Cooperative Oncology Group performance status of 0 or 1. The key exclusion criteria included a lack of baseline data; chemotherapy, radiotherapy, or surgery for MSTS prior to treatment; an interruption initiated by a non-drug itself in the NPI or NIV regimen; symptomatic central nervous metastasis; severe metabolic disorders (i.e., hyperthyroidism and hypophysoma); drug abuse; psychosis, or cognitive disorder.

### Outcomes and assessments

PD-L1 expression on biopsy was assessed by immunochemistry using the anti PD-L1 monoclonal antibody, which was consistent with the previous description [13]. Positive PD-L1 expression was defined as staining of the plasma membrane in more than 1% of tumour cells [14]. OS was defined as the time from first dose to the date of death; PFS, from first dose to progression or death due to any cause, whichever came first. Drug toxicity analysis was performed using the approved product label for all evaluable patients who had undergone NPI or NIV treatment. Tumour responses were judged every 6 weeks until progression or drug interruption per RECIST v1.1. The RECIST was measured retrospectively. AEs were coded per the Medical Dictionary for Regulatory Activities (v 19.0). AE severity was graded per the Common Terminology Criteria for Adverse Events, v4.0 [7]. Follow-up was conducted every 2 months.

### Statistical analysis

We used the chi-square test for categorical data; continuous variables were compared with Student t-test for normally distributed variables and Mann-Whitney U test for non- normally distributed variables. Median follow-up was estimated using the reverse Kaplan-Meier method. OS and PFS were estimated per the Kaplan-Meier method. Hazard ratios (HRs) were estimated by a Cox proportional hazard model with 95% confidence intervals (CIs). All *p* values were two-sided with the level of significance set to 0.05. We executed data analyses using SPSS v 26.0 (IBM, Inc., NY, USA).

## Results

### Comparison of baseline data

**Fig. 1:**
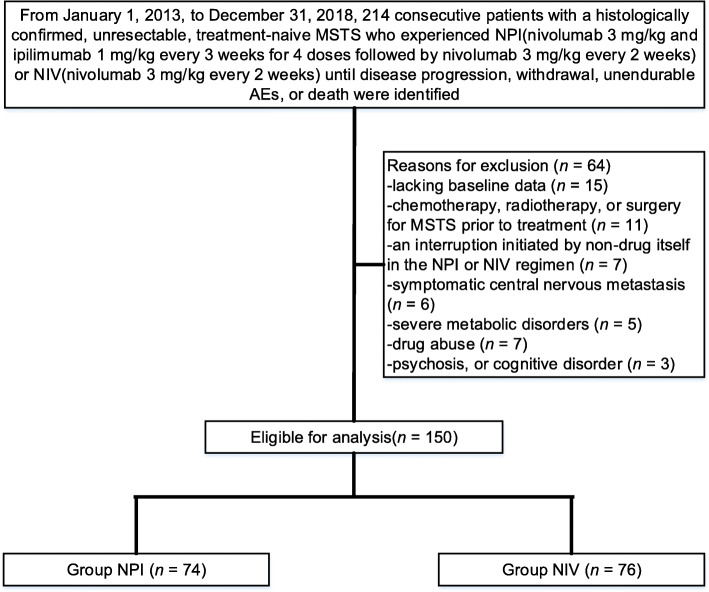
Flow diagram demonstrating the methods used for the identification of this study to retrospectively assess the efficacy and safety of nivolumab plus ipilimumab (NPI) versus nivolumab alone (NIV) in individuals with untreated programmed death-ligand 1 (PD-L1) positive MSTS

A total of 214 patients with treatment-naive PD-L1 positive MSTS were reviewed, 64 of whom were deemed to be ineligible according to our criteria, leaving 150 patients (NPI: *n*=74, median age 35 years [21.2–51.8] and NIV: *n*=76, median age 34 years [23.8–57.3]) who were finally included for eligibility (Fig. 1). Of the 150 evaluable patients whose PD-L1 expression was validated, 150 (100%) suffered PD-L1–positive tumours. Baseline data reported here were well balanced between groups (Table 1). Patients underwent a median 6 drug cycles (IQR 2.0–8.0), with a median follow-up period of 16.0 months (IQR 14.4–18.5) after targeted intervention.

**Table 1:** Patient demographics and disease characteristics at baseline

Variable	NPI (*n*=74)	NIV (n=76)	*p*-value
Age, years			
Median (range)	35 (21.2–51.8)	34 (23.8–57.3)	0.105
Sex, n (%)			0.729
Male	42 (56.8)	41 (53.9)	
Female	32 (43.2)	35 (46.1)	
BMI, kg/m^2^			
Median (range)	25.7 (17.1–41.3)	25.4 (15.6–43.7)	0.256
ECOG performance status, n (%)			0.619
0	40 (54.1)	38 (50.0)	
1	34 (45.9)	38 (50.0)	
Sarcoma types^a^, n (%)			0.764
Non-uterine leiomyosarcoma	43 (58.1)	40 (52.6)	
Liposarcoma^b^	20 (27.0)	22 (28.9)	
Synovial sarcoma	11 (14.9)	14 (18.4)	
Three major types of liposarcoma			0.078
Atypical lipoma	9 (12.1)	11 (14.5)	
Myxoid liposarcoma	10 (13.5)	9 (11.8)	
Pleomorphic liposarcoma	1 (1.4)	2 (2.6)	
Histological grade, n (%)			0.504
G1 (well differentiated)	31 (41.9)	37 (48.7)	
G2 (moderately differentiated)	27 (36.5)	21 (27.6)	
G3 (poorly differentiated)	16 (21.6)	18 (23.7)	
TMB^c^ status (per Mb), n (%)			0.716
TMB-High (> 5)	45 (60.8)	44 (57.9)	
TMB-Low (0–5)	29 (39.2)	32 (42.1)	
Duration of treatment (months)			
Median (range)	3 (1.5–4.4)	3 (1.6–4.7)	0.317
Number of metastatic sites, n (%)			0.583
3	12 (16.2)	10 (13.2)	
> 3	51 (68.9)	58 (76.3)	
Unknown	11(14.9)	8 (10.5)	

^a^Based on a central review of pathology; ^b^Primary liposarcomas were located in the lower extremity (11%), upper extremity (6%), the trunk wall (11%), the retroperitoneum (64%), and the head and neck (8%); ^c^defined as the number of somatic coding base substitution and indels per megabase of genome. *NPI* nivolumab plus ipilimumab, *NIV* nivolumab, *BMI* body mass index, *ECOG* Eastern Collaborative Oncology Group, *TMB* tumour mutation burden

### Comparison of efficacy

**Fig. 2:**
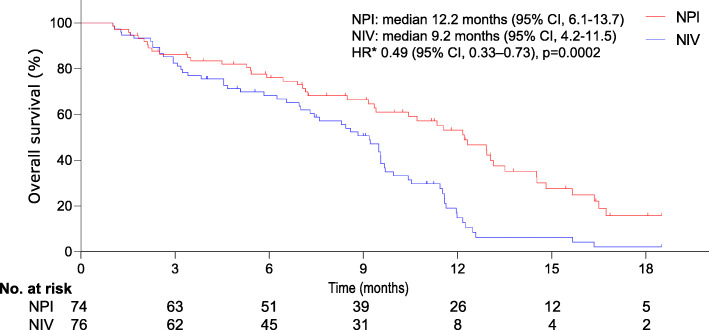
Kaplan–Meier curves for overall survival. The median overall survival was 12.2 months (95% CI, 6.1–13.7) and 9.2 months (95% CI, 4.2–11.5) for the NPI and NIV groups, respectively (HR 0.49, 95% CI, 0.33–0.73; *p*=0.0002). Significant differences were detected in the overall survival between groups. *The hazard ratio was calculated using a Cox proportional hazards model, with age, the sarcoma types, the number of metastatic sites, and the ECOG performance status as covariates and NPI or NIV therapy as the time-dependent factor

**Fig. 3:**
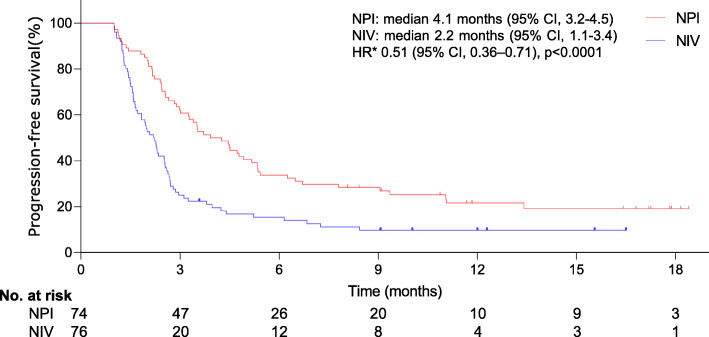
Kaplan–Meier curves for progression-free survival. The median progression-free survival was 4.1 months (95% CI, 3.2–4.5) and 2.2 months (95% CI, 1.1–3.4) in the NPI or NIV groups, respectively (HR 0.51, 95% CI 0.36–0.71; *p*< 0.0001). Statistically significant differences were detected in the progression-free survival between groups. *The hazard ratio was calculated using a Cox proportional hazards model, with age, the sarcoma types, the number of metastatic sites, and the ECOG performance status as covariates and NPI or NIV therapy as the time-dependent factor

A significant difference was observed in the proportion of patients with a confirmed response rate (13% [95% CI, 1–17] for NPI vs. 7% [95% CI, 1–11] for NIV). At the final analysis, individuals with unresectable, treatment-naive MSTS who experienced NPI had a median OS of 12.2 months (95% CI, 6.1–13.7), which was significantly longer than that of patients receiving NIV (9.2 months, 95% CI, 4.2–11.5). The distinction in OS corresponded to an HR of 0.49 (95% CI, 0.33–0.73, *p*=0.0002) (Fig. 2). A significant difference was also detected in median PFS (4.1 months [95% CI, 3.2–4.5] for NPI vs. 2.2 months [95% CI, 1.1–3.4] for NIV; HR 0.51, 95% CI, 0.36–0.71; *p*< 0.0001), as shown in Fig. 3. The survival advantage of NPI versus NIV was more dramatic.

### Adverse events

All 150 individuals included who underwent NPI or NIV treatment suffered AEs of any grade. The key grade 3–5 AEs observed were shown in Table 2. At the time of this analysis, key grade 3–5 AEs occurred more frequently in the NPI group than in the NIV group (94 [72.9%] for NPI vs. 35 [27.1%], *p*< 0.001). Discontinuation of NPI or NIV associated with AEs occurred in six (14%) of 74 patients in the NPI group and one (2%) of the 76 patients in the NIV group. Hyponatraemia occurred in 9 patients (12.2%) in the NPI group and 1 (1.3%) in the NIV group (*p*=0.008). Hypotension was more frequent in the NPI group than in the NIV group (8 [10.8%] vs. 0 [0.0%], respectively, *p*=0.003). Significant differences were also observed in terms of increased aspartate aminotransferase (5 [6.8%] for NPI vs. 0 [0.0%] for NIV, *p*=0.021), dyspnoea (6 [8.1%] for NPI vs. 0 [0.0%] for NIV, *p*=0.011), nervous system disorders (8 [10.8%] for NPI vs. 1 [1.3%] for NIV, *p*=0.014), urinary tract infection (4 [5.4%] for NPI vs. 0 [0.0%] for NIV, *p*=0.040), and ≥ 2 AEs in a patient (23 [31.1%] for NPI vs. 12 [15.8%] for NIV, *p*=0.027). The most frequent grade ≥3 AE was anaemia, which occurred in 9 patients (12.2%) in the NPI group and 8 (10.5%) in the NIV group (*p*=0.752). In addition, no significant differences were detected in regard to colonic perforation, increased creatinine, dehydration, vaginal fistula, increased lipases, lung infection, nausea, or skin infection. Drug-related deaths did not occur in either group.

**Table 2:** Comparison of the incidence of key drug-related grade ≥3 AEs at final follow-up

Event, n (%)	NPI (*n*=74)	NIV (*n*=76)	*p*-value
Anaemia	9 (12.2)	8 (10.5)	0.752
Aspartate aminotransferase increased	5 (6.8)	0 (0.0)	0.021^a^
Colonic perforation	0 (0.0)	1 (1.3)	1.000
Creatinine increased	2 (2.7)	2 (2.6)	0.978
Dehydration	3 (4.1)	1 (1.3)	0.298
Dyspnoea	6 (8.1)	0 (0.0)	0.011^a^
Hyponatraemia	9 (12.2)	1 (1.3)	0.008^a^
Hypotension	8 (10.8)	0 (0.0)	0.003^a^
Vaginal fistula	3 (4.1)	0 (0.0)	0.076
Lipases increased	4 (5.4)	4 (5.3)	0.969
Lung infection	3 (4.1)	3 (3.9)	0.973
Nausea	4 (5.4)	2 (2.6)	0.386
Nervous system disorders	8 (10.8)	1 (1.3)	0.014^a^
Skin infection	3 (4.1)	0 (0.0)	0.076
Urinary tract infection	4 (5.4)	0 (0.0)	0.040^a^
≥ 2 AEs in one patient	23 (31.1)	12 (15.8)	0.027^a^

^a^Statistically significant. *AEs* adverse events, *NPI* nivolumab plus ipilimumab, *NIV* nivolumab

## Discussion

This study shows that for treatment-naive PD-L1 positive MSTS, the superiority of NPI over NIV in terms of survival benefit tends to be positive, which is in line with previous reports involving individuals with untreated MSTS [7, 11]. Safety profiles were consistent with those of other solid tumours (i.e., melanoma) [15].

Our findings might provide a confirmation that NPI improves survival for individuals with untreated MSTS. In a multicentre, open-label, non-comparative, randomized phase 2 study [7], 85 eligible patients with metastatic sarcoma who were treated using NPI (42 cases) or NIV (43 cases) showed a median PFS of 4.1 months (2.6–4.7) and 1.7 months (95% CI 1.4–4.3), respectively; the median OS was 14.3 months (9.6–not reached) with NPI and 10.7 months (5.5–15.4) with NIV. These findings may be instructive when placed in the context of presently accessible treatment options for individuals with untreated MSTS [16]. The classic treatment for MSTS tends to be based on cytotoxic chemotherapy, with first-line therapy predictably accomplishing objective responses in 15–18% of individuals, with a median PFS of 4–6 months [7, 17]. Activity beyond the first-line options tends to decline, with less than 10% of individuals reaching objective responses and a median PFS of 1–4 months [18]. In the current review, the choice of NPI or NIV as a monotherapy, regardless of its combination with cytotoxic chemotherapy, may have a negative impact on survival. However, a key challenge with MSTS is that well-established protocols for management tend to be lacking, and in the absence of distinguishable signs or symptoms identifiable by the clinicians, diagnosis tends to be difficult; indeed, once diagnosed with STS, the patient is generally in the late stage of the disease, ultimately leading to reduced survival [16, 19].

A double-blind trial [20] involving 142 patients with treatment-naive MSTS showed that meaningfully longer PFS was detected with NPI than with ipilimumab monotherapy (not reached vs. 4.4 months [95% CI 2.8–5.7]; HR 0.40, 95% CI 0.23–0.68, *p*< 0.001). The response rate associated with NPI in their study (61%) was higher than with NIV (61% vs. 40%) as first-line therapy in such individuals. The response rate of the combination therapy in our study was also higher than the rate detected in published trials involving anti–PD-1 agent-based monotherapy (i.e., pembrolizumab) [21, 22]. Nevertheless, a comparison of the efficacy of NPI and anti-PD-1 monotherapy may be challenging due to differences in the baseline data of individuals among the studies. The PFS and OS seen with NPI in our review are in accordance with those reported elsewhere [7, 11, 23], with the primary endpoint occurring by the time of the final tumour evaluation and, in a host of patients, OS being prolonged as follow-up continued regardless of termination of treatment, which might be elucidated by the fact that the individuals included in this review were diagnosed with treatment-naive MSTS.

Antibodies against PD-1 or PD-L1 have a positive effect in blocking tumour immune evasion and inducing tumour regression in STS [7, 24]. Previous reports [7, 11, 25, 26] of PD-L1 expression have shown that STS is potentially responsive to PD-1/PD-L1 blockade intervention in STS patients with PD-L1 positivity. The survival benefit of NIV monotherapy is inconsistent with presently existing chemotherapy-based untargeted therapies [7, 11]. Furthermore, in previous trials [8, 23], NIV patients failed to meet the predetermined primary outcome of completing responses in more than 13% of cases to sustain activity in their setting, which could exclude extended trials for heavily treated, unselected patients with MSTS [11, 16]. NPI patients met this predetermined primary outcome among those unselected patients with MSTS [24, 26, 27]. The proportion of NPI patients reaching an objective response appeared to be 16%, approximating that realized via accepted chemotherapy-based management [26, 27]. Additionally, an objective response of approximately 16% in 38 patients is in accordance with FDA-approved chemotherapy regimens, theoretically favouring future trials of NPI not only as second-line management in patients with MSTS but also as a first-line treatment option [7, 26]. Although patients undergoing a treatment regimen approved by the FDA exhibited a median OS of 26.5 months (95% CI, 20.9–31.7), their data might not truly reflect survival in an open-label phase 1b and randomized phase 2 trial [28]. However, the OS seen with NPI in this study is promising and indicates the potential to improve survival in patients with MSTS.

The safety results associated with NPI and NIV were in accordance with prior studies [7, 26]. In this review, NIV tended to be better tolerated, with a lower rate of AEs compared with NPI. The rate of grade 3–5 AEs among individuals experiencing NIV was 27.1%. The safety results contrasted with the results reported in a previous study [7], where the dose of NIV was higher than the recommended dose, and a higher percentage of individuals suffered from grade 3–5 AEs. Adopting a lower dose of NIV could potentially improve the rate of AEs. Remarkably, the proportion of grade 3–5 AEs described in this review for NIV was lower than that of cytotoxic drugs in the current setting.

Several limitations should be recognized in this review. First, this study is a retrospective study, with its inherent shortcomings and some potential confounding variables (i.e., potential comorbidities and complications, some patients who were followed up by telephone), which reduces the reliability of the conclusion. Second, the sample size of this retrospective review is limited, which restricts the generalizability of the results to some extent. Third, gene mutation types are not retested when the disease progresses, and drug resistance mutations during treatment have not been tested for each individual. Therefore, when drug resistance mutations appear in some individuals, the power of this study to reach a reliable conclusion is weakened. Fourth, the current research objects were collected from different tertiary medical centres, and there might be some differences in the diagnosis process of these medical institutions. Nevertheless, these research objects are coded and combined through standardized methods, which guarantees the reliability of the research conclusions.

## Conclusion

The results reported in the current review reiterate an increasing body of evidence showing that for individuals with treatment-naive PD-L1 positive MSTS who undergo treatment with NPI or NIV, NPI seems to be less tolerated but has a greater survival advantage as the primary therapy than NIV. Our findings might underline the promise of combined checkpoint inhibition in the current setting. These data should be validated prospectively in subsequent analyses of larger cohorts with treatment-naive MSTS.

## Data Availability

The datasets used during the current study are available from the corresponding author on reasonable request.

## Abbreviations

MSTS: Metastatic soft tissue sarcomas
NPI: Nivolumab plus ipilimumab
NIV: Nivolumab
AEs: Adverse events
OS: Overall survival
PFS: Progression-free survival
IQR: Interquartile range
PD-1: Programmed death 1
CIs: Confidence intervals
HRs: Hazard ratios
RECIST: Response Evaluation criteria in solid tumours
ECOG PS: Eastern Collaborative Oncology Group performance status
PD-L1: Programmed death-ligand 1
TMB: Tumour mutation burden
